# Cloning and Characterization of *tesk1*, a Novel Spermatogenesis-Related Gene, in the Tongue Sole (*Cynoglossus semilaevis*)

**DOI:** 10.1371/journal.pone.0107922

**Published:** 2014-10-01

**Authors:** Liang Meng, Ying Zhu, Ning Zhang, Wanjun Liu, Yang Liu, Changwei Shao, Na Wang, Songlin Chen

**Affiliations:** 1 College of Marine Life Science, Ocean University of China, Qingdao, PR China; 2 Yellow Sea Fisheries Research Institute, Chinese Academy of Fishery Sciences, Qingdao, PR China; Temasek Life Sciences Laboratory, Singapore

## Abstract

Testis-specific protein kinase 1 (Tesk1) is a serine/threonine kinase with unique structural features. In the present study, we cloned and characterized the *tesk1* gene of tongue sole, *Cynoglossus semilaevis*. The full-length *tesk1* cDNA consists of 1,672 nucleotides, encoding a 331 amino acid polypeptide with a characteristic structure composed of an N-terminal kinase domain and a C-terminal proline-rich domain. The *tesk1* genomic sequence contains eight exons and seven introns. Real-time quantitative PCR revealed that *tesk1* mRNA is expressed predominantly in the testis, though the level of expression varied throughout development. We used *in situ* hybridization to show that *tesk1* mRNA is expressed in the spermatids of males and pseudo-males, but not in triploid males. Our results suggest that tongue sole Tesk1 may play a role in spermatogenesis.

## Introduction

Protein kinases play a pivotal role in intracellular signal transduction systems, particularly those involved in the regulation of cell metabolism, proliferation and differentiation. They are divided into two broad categories based on their substrate specificity. Tyrosine kinases exhibit specificity toward the tyrosine residue, whereas serine/threonine kinases exhibit substrate specificity toward serine and threonine residues [1], [2], [3]. Testis-specific protein kinase 1 (Tesk1) is a member of the latter group and was first identified in the testis of rats and humans [4]. It has an N-terminal protein kinase domain and a C-terminal extension rich in proline residues. High levels of *tesk1* mRNA expression and protein production have been detected in the testicular germ cells of rats between the late pachytene spermatocyte and round spermatid stages, but not in rat somatic cells (e.g., Sertoli and Leydig cells). Expression of *tesk1* mRNA and protein at specific stages in testicular germ cell development suggests that this kinase plays a role in spermatogenesis, particularly during meiosis and/or early spermiogenesis [5].

Tongue sole (*Cynoglossus semilaevis*) is an economically important fish that is widely cultured in China. According to information statistics, about 10,580 tons of tongue soles were produced in China in 2012. Additionally, this species is a promising model for the study of sex determination mechanism because it exhibits sexual dimorphism in its growth, with females growing 2–4 times faster than males [6]. Moreover, the percentage of phenotypic males in a given culture population is higher than that of females because of natural sex reversal [6], [7]. These two traits result in higher numbers of small adult males, which have less commercial value than the larger females. This has resulted in a desire to develop methods for increasing the ratio of female tongue sole in culture. To address this issue, Chen et al. (2009) attempted to develop an artificial gynogenetic population to produce all-female fish, but achieved limited success [8].

Tongue sole exhibits ZW type sex determination characterized by the male homogametic (ZZ) and female heterogametic (ZW) sex chromosomes [9]. Pseudo-males are phenotypic males derived from the ZW genetic female by sex reversal. Theoretically, the crossing of a ZW female and a ZW pseudo-male should produce 50% ZW females, 25% ZZ males and 25% WW super-females; however, our previous experimental results were inconsistent with the hypothesis [10]. Instead, the ratio of genetic females to genetic males was nearly 50% when using a pseudo-male as the male parent. Interestingly, the rate of sex reversal was higher in the offspring of pseudo-males than normal males [10], [11]. Moreover, analysis of the genotype of fertile sperm from ZW pseudo-males using sex chromosome-specific microsatellite markers revealed no W sperm [10].

The whole-genome sequencing of tongue sole demonstrated that there is a differentiation of the genes between Z and W chromosomes, with 926 functional genes on the Z chromosome, but only 317 on the W chromosome [10] Additionally, the ubiquitin E3 ligase gene *neurl3-like*, which is involved in spermatogenesis, is only present on the Z chromosome [10]. Because of this, we hypothesized that key genes related to spermatogenesis are only distributed on the Z chromosome, and that the absence of W sperm may result from the lack of these genes on the W chromosome. Therefore, it is important to identify such spermatogenesis-related genes in tongue sole. Although *tesk1* was previously shown to be a spermatogenesis-related gene in humans and mice [5], no study has yet investigated the role of *tesk1* in teleost spermatogenesis.

In the present study, we cloned and characterized *tesk1* from tongue sole, and found it to be a Z chromosome-specific gene, which is likely to play an important role in spermatogenesis. This is the first report about the involvement of *tesk1* in the spermatogenesis of teleosts.

## Materials and Methods

### Ethics statement

All experimental animal protocols were approved by the Yellow Sea Fisheries Research Institute’s animal care and use committee. All tissues were removed under MS222 anesthesia, and all efforts were made to minimize fish suffering.

### Experimental fish preparation

*C. semilaevis* were purchased from the Haiyang High-Tech Experimental Base (Haiyang, China). Artificial triploids of *C. semilaevis* were generated using hydraulic pressure shock as described by Chen et al. [12]. Triploidy was identified by karyotyping and flow cytometry analysis of DNA relative content [12]. Six samples of each category, including the male, female, pseudo-male and triploid male, were prepared for the present study.

### Tissue collection

Tissues (heart, liver, kidney, intestine, spleen, skin, muscle, brain and gonads) were collected and quick-frozen in liquid nitrogen. The gonad was removed from one side of the fish at different stages of maturation and immediately frozen in liquid nitrogen until use. The gonad from the other side was also removed and divided into two sections: one was placed in Bouin’s fixative for histological analysis and the other was placed in 4% paraformaldehyde for *in situ* hybridization (ISH). Finally, the fin was collected and stored in 100% ethanol for DNA extraction to determine the genetic sex.

### Identification of phenotypic and genetic sex

To identify the phenotypic sex, gonadal histology of all experimental samples was carried out as previously described [13]. The genetic sex was determined by following the methods of Chen et al. [14]. Briefly, genomic DNA was extracted from each tongue sole fin using phenol-chloroform extraction [14] with RNase I (EC: 3.1.4.22). The sex-specific simple sequence repeat (SSR) markers Cse-SSR1F and Cse-SSR1R (Table S1) were used for PCR amplification as previously described [14]. Using this pair of primers, a 206 bp band was amplified from ZZ males and ZZZ triploid males, and two bands of 206 and 218 bp were amplified from ZW females and ZW pseudo-males.

### Cloning of *C. semilaevis* tesk1 full length cDNA

Total RNA was extracted using TRIzol Reagent (Invitrogen, Carlsbad, CA, USA) according to the manufacturer’s instructions. To carry out rapid amplification of cDNA ends (5′ and 3′-RACE), two specific primers (TEGSP5 and TEGSP3, Table S1) were designed based on the *tesk1* partial cDNA sequence from tongue sole genome sequencing. We conducted 5′-RACE and 3′-RACE using the SMART RACE cDNA Amplification Kit (Clontech Inc., Mountain View, CA, USA) following the manufacturer's instructions. Touchdown PCR was performed as follows: five cycles of 94°C for 30 s and 72°C for 3 min; five cycles of 94°C for 30 s, 70°C for 30 s, and 72°C for 3 min; 25 cycles of 94°C for 30 s, 68°C for 30 s, and 72°C for 3 min; then 72°C for 10 min as an elongation. The amplified products were electrophoresed on a 1% agarose gel. Bands of expected size were excised from the gel and purified with a Zymoclean Gel DNA Recovery Kit (ZYMO Research, Orange, CA, USA). Purified fragments were cloned into a pMD18-T vector (TaKaRa, Dalian, China), propagated in *Escherichia Coli* TOP10 (Tiangen, Beijing, China), and positive clones were sequenced on an ABI 3730xl DNA analyzer (Applied Biosystems, Foster City, CA, USA) using SP6 or T7 primers.

### Amplification of *tesk1* genome sequence

The *tesk1* cDNA sequence was analyzed by bioinformatics and three pairs of primers were designed for genomic amplification: TEG1S and TEG1A; TEG2S and TEG2A; and TEG3S and TEG3A (Table S1). Cloning and sequencing were conducted as described above. The obtained sequences were assembled using the Lasergene DNASTAR software package.

### Bioinformatic sequence analysis

Sequences were analyzed according to nucleotide and protein databases using the BLAST website (http://www.ncbi.nlm.nih.gov/BLAST/) [15]. The coding sequence was predicted using the NCBI open reading frame (ORF) finder (http://www.ncbi.nlm.nih.gov/gorf/orfig.cgi), and the gene structure was also analyzed (http://gsds.cbi.pku.edu.cn). The conserved domain was analyzed by conducting an NCBI conserved domain search (http://www.ncbi.nlm.nih.gov/structure). The molecular weight of the protein was deduced (http://www.bio-soft.net/sms/index.html), and signal peptides were predicted using Signal 3.0 [16] (http://www.cbs.dtu.dk/services/SignalP). Multiple alignments of amino acid sequences were performed using Clustal W 2.0 [17]. Phylogenetic relationships were deduced using Mega 4.0 software [18].

### Real-time quantitative PCR

First strand cDNA synthesis was carried out using a PrimeScript RT reagent Kit with gDNA Eraser (TaKaRa). Quantitative (q) PCR was performed in 20 µL reactions in a 7500 ABI real-time PCR system (Applied Biosystems). The reaction was carried out as described in the manufacturer's instructions using 10 µL SYBR Premix Ex Taq (2×), 0.4 µL of TE-RTF and TE-RTR (Table S1), 0.4 µL ROX reference dye II and 1 µL cDNA. The PCR amplification procedure was as follows: 95°C for 30 s, then 40 cycles of 95°C for 5 s, and 60°C for 34 s. We conducted a disassociation curve analysis to determine target specificity. *β-actin* was used as the internal control [19]. Each sample was analyzed in triplicate and at least three samples were processed.

The concentration of cDNA in each sample was reflected by the Cq values, which were compared using the relative quantification method and 7500 system SDS software (Applied Biosystems). Data were log-transformed and differences between groups were tested using one way ANOVA followed by Duncan multiple comparison tests using SPSS 18.0 (IBM, New York, NY, USA). Significance was set at *p*<0.05.

### *In situ* RNA hybridization

To synthesize RNA probes, we designed the primers pair: TESK-ISH-F and TESK-ISH-R (Table S1). A 241 bp cDNA fragment located in the tongue sole *tesk1* ORF domain was amplified and inserted into a pBluescriptIISK+ plasmid. The recombinant plasmid was linearized with *EcoR* V and *Sal* I and used as a template for transcription with T7 or T3 RNA polymerase for antisense and sense control probes, respectively. DIG-NTP was used to label RNA probes. The *in situ* hybridization was performed using the labelled probes as previously described [10], and three samples were processed.

### Fluorescence *in situ* chromosome hybridization

Chromosomal localization of tongue sole *tesk1* was performed by fluorescence *in situ* hybridization (FISH). Three *C. semilaevis* weighing about 800 g were held at 25°C for 24 h then injected with colchicine. About 3 h after injection, they were killed and the head–kidney was removed. Metaphase chromosomes were prepared from the cells of the head–kidney as described by Shao et al [9]. A BAC clone Hind017D15 [20] containing *C. semilaevis tesk1* was isolated using the *tesk1* genomic sequence, labeled with biotin-16-dUTP using a Nick-Translation System (Roche, Branford, CT, USA) and used as a hybridization probe. FISH conditions have been described previously [10]. In brief, metaphase chromosomes were hybridized with the probe at 37°C for 24 h in a solution containing 2 × SSC (0.15M NaCl, 15 mM sodium citrate, pH 7.0), 50% formamide, 10% dextran sulfate, and 2 mg/ml bovine serum albumin. Signal detection and amplification were performed using sheep-anti-digoxigenin and FITC-Donkey-anti-sheep (Dig-Nick Translation Mix, Roche, 11745816910). FISH staining was performed with propidium iodide. Image capture was carried out using a NIS-element fluorescence microscope (Nikon, Tokyo, Japan) and then analyzed by the LUCIA system and Adobe Photoshop software.

## Results

### Cloning and characteristics of tongue sole *tesk1*

A 906 bp fragment corresponding to no. Cse_R016807 was obtained and identified as tongue sole *tesk1* by gene prediction and annotation based on tongue sole genome sequencing [10]. Two additional fragments of 512 and 540 bp were produced by 5′ RACE and 3′ RACE, respectively. These three fragments were assembled into a 1,672 bp tongue sole *tesk1* full length cDNA (GenBank accession no. KF939086). This complete cDNA contains a 241 bp 5′ untranslated region (UTR), a 996 bp ORF, and a 435 bp 3′ UTR with a single typical polyadenylation signal (AATAAA) between nucleotides 1,628–1,633. The 7,271 bp tongue sole *tesk1* genomic sequence (GenBank accession no. KJ679501) contains eight exons and seven introns. Each intron exhibits a typical intron splice motif at the 5′(GT) and 3′(AG). A 54 bp microsatellite sequence was predicted to have (CT)_27_ in the fourth intron (Fig. S1). The *tesk1* gene resides on the Z chromosome (5029371–5036642, sense strand) [10].

The ORF encodes a 331 amino acid protein with a predicted molecular weight of 37.81 kDa. There was no evidence of a signal peptide. Based on conserved domain searching, an N-terminal kinase domain composed of several active sites was detected, and a proline-rich domain was identified at the C-terminal (Fig. S1).

### Alignment and phylogenetic analysis

**Figure 1:**
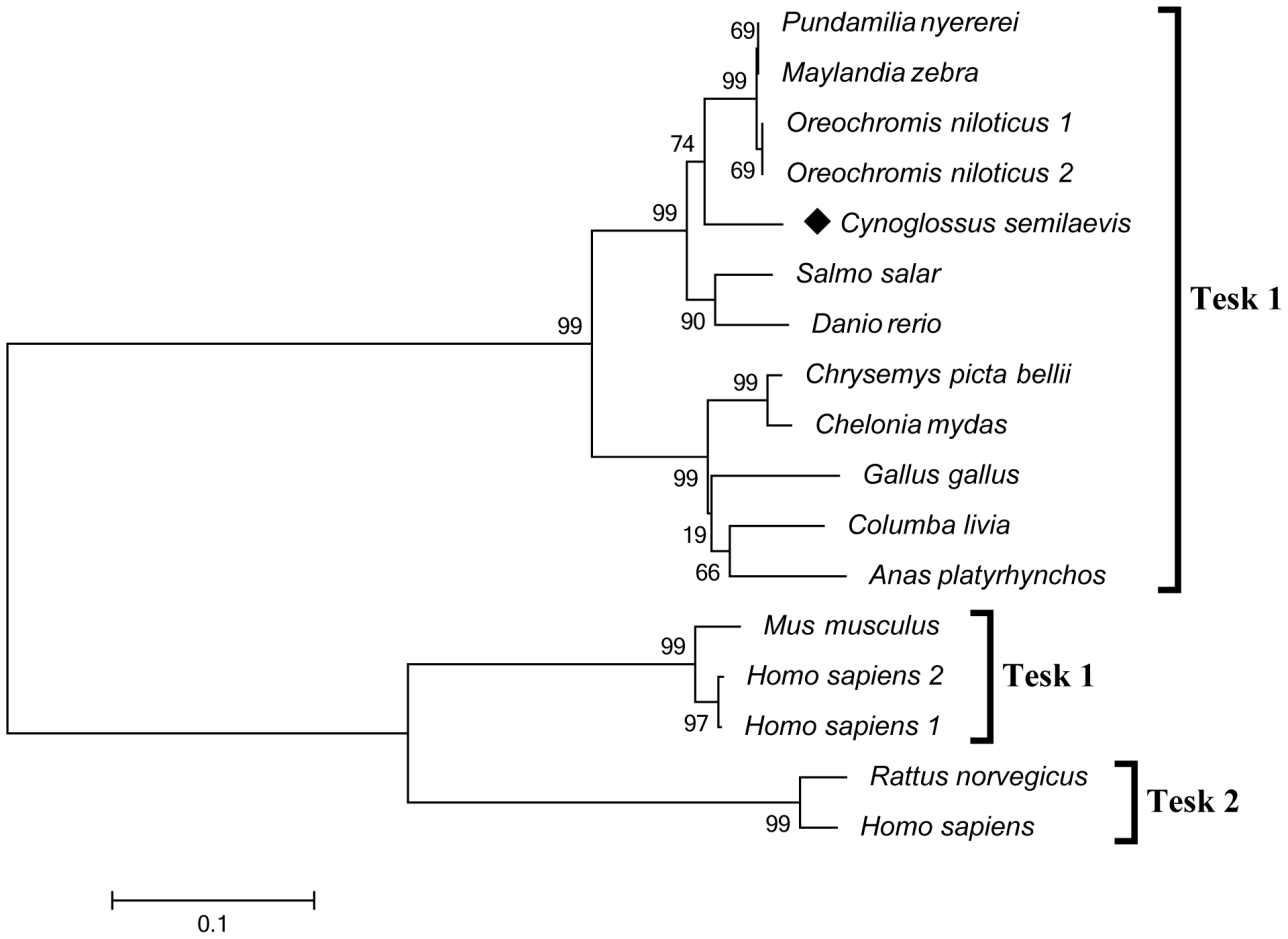
Phylogenetic analysis of Tesk protein sequences. A phylogenetic tree was constructed with the neighbor-joining algorithm in MEGA 4.0. Dendrogram graphically showed the relations for different organisms based on the amino acid sequence. The relative genetic distances are indicated by the scale bar and the branch lengths. Protein sequences used in this analysis: *Pundamilia nyererei* Tesk1 (XP_005745859), *Maylandia zebra* Tesk1 (XP_004565879), *Oreochromis niloticus* Tesk1 isoform1 (XP_003459044), *Oreochromis niloticus* Tesk1 isform2 (XP_005464337), *Salmo salar* Tesk1 (ACI33724), *Danio rerio* Tesk1 (NP_001083043), *Gallus gallus* Tesk1 (XP_003642315), *Chrysemyspicta bellii* Tesk1 (XP_005291483), *Chelonia mydas* Tesk1 (EMP299900), *Columba livia* Tesk1 (EMC88537), *Anas platyrhynchos* Tesk1 (EOB04897), *Mus musculus* Tesk1 (NP_035701), *Homo sapiens* Tesk1 isoform1 (NP_006276), *Homo sapiens* Tesk1 isoform2 (BAA09459), *Rattus norvegicus* Tesk2 (NP_596887), *Homo sapiens* Tesk2 (NP_009101).

Alignment of the amino acid sequence encoded by tongue sole *tesk1* was performed based on a BLASTp search of the sequence in other species. The Tesk1 protein of tongue sole shows a high degree of identity (87–94%) with its orthologs from other teleost species, such as *Danio rerio* (NP_001083043), *Salmo salar* (ACI33724), *Oreochromis niloticus* (XP_003459044 and XP_005464337), *Maylandia zebra* (XP_004565879) and *Pundamilia nyererei* (XP_005745859). It displays a lower degree of identity (about 50%) with mammals compared with fish, birds, and reptiles. A phylogenetic tree was constructed based on the amino acid sequences to study the relationship between tongue sole Tesk1 and that of other species. This grouped fish into one clade, while birds and reptiles were grouped into a separate clade. Tesk1 and Tesk2 of mammals were grouped into another clade (Fig. 1).

### Tissue distribution of *tesk1*

**Figure 2:**
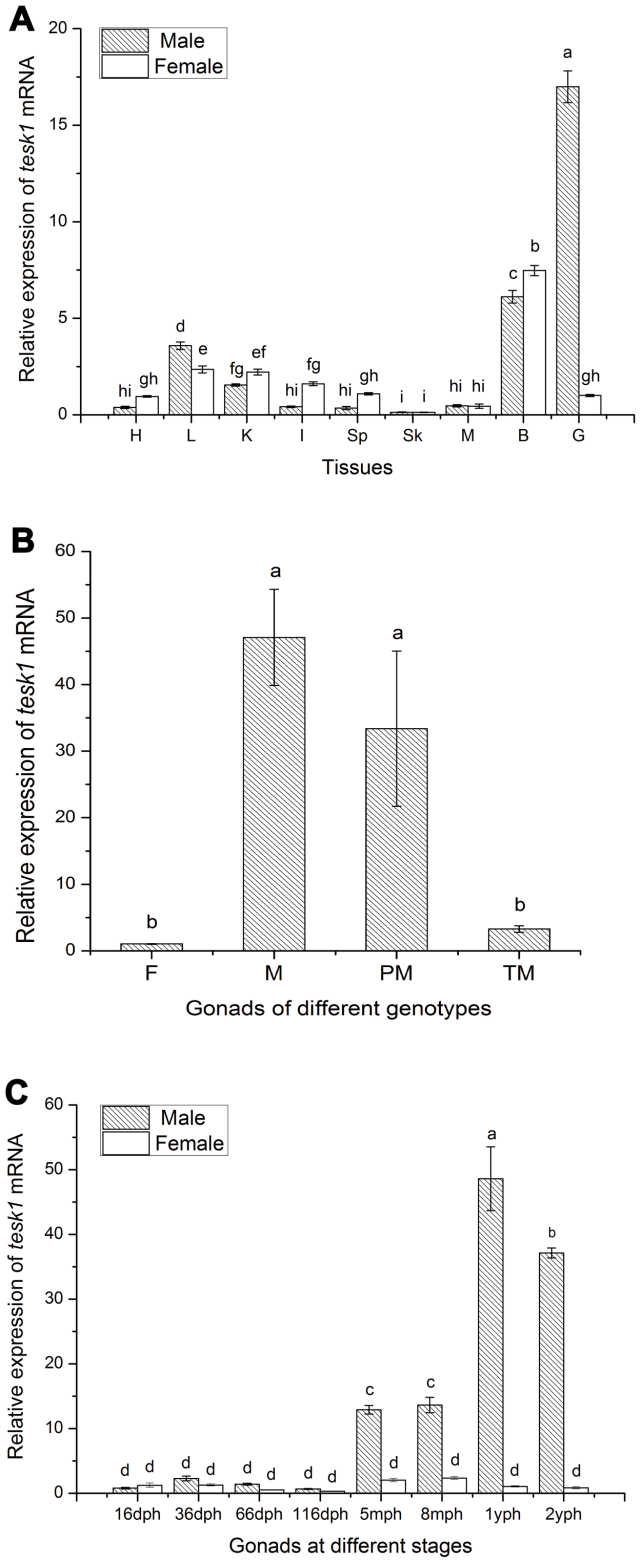
Real-Time quantitative PCR analysis of *tesk1* of *C.semilaevis*. (A) The expression of *tesk1* in various tissues of tongue sole. H: heart, L: liver, K: kidney, I: intestine, SP: spleen, SK: skin, M: muscle, B: brain, G: gonad. (B) The expression of *tesk1* in gonads of different genotypes. F: female, M: male, PM: pseudo-male, TM: triploid male. (C) The expression of *tesk1* at different developmental stages of the gonads. The *tesk1* mRNA amount was normalized to the *β-actin* transcript level. The data was analyzed by one-way ANOVA followed by Duncan comparison tests using SPSS 18.0. Bars represent the triplicate mean±SE from three separate individuals (n = 3). Bars with different letters differed with statistical significance (*p<0.05*).

To analyze tissue expression levels of tongue sole *tesk1*, real-time qPCR was conducted using total RNA from a range of tissues in 2-years-old male and female fish. The mRNA expression of *tesk1* was detected in a variety of tissues (Fig. 2A), being highest in the testes and very low in the ovaries. Low levels of expression were also detected in the brain, liver, and kidney, and extremely low levels were found in remaining tissues.

### *Tesk1* expression in the gonads of male, pseudo-male, triploid male and female tongue sole

**Figure 3:**
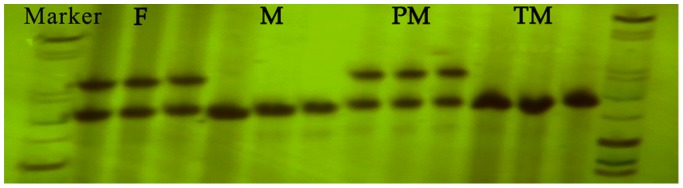
Identification of genotype of *C.semilaevis*. F: female, M: male, PM: pseudo-male, TM: triploid male. One 206 bp band corresponding to diploid male (ZZ) and triploid male (ZZZ), two bands (206 bp and 218 bp) corresponding to diploid female (ZW) and diploid pseudo-male (ZW). Triploidy has been identified by flow cytometry analysis of DNA relative content and karyotyping.

Sex-specific SSR markers were used to determine the genetic sex [14], identifying ZZ males and ZW pseudo-males (Fig. 3). Triploid males were also identified with a ZZZ genotype.

Real-time qPCR revealed very low levels of *tesk1* expression in the testes of ZZZ triploid males and the ovaries of females. By contrast, high levels of *tesk1* expression were detected in ZW pseudo-males, though this varied greatly between individuals (Fig. 2B).

### *Tesk1* expression pattern in the developing male gonads

Extremely low levels of *tesk1* expression were detected in the gonads of fry at 16, 36, 66, and 116 days of age. *tesk1* transcription increased significantly in the testes between 5 months and 2 years after hatching. In particular, the expression of *tesk1* increased significantly 1 year after hatching in the testes of males. By contrast, there was no change in the level of *tesk1* mRNA expression in the ovaries of females between 116 days and 2 years after hatching (Fig. 2C).

### Cellular localization of *tesk1* mRNA expression in the gonads

**Figure 4:**
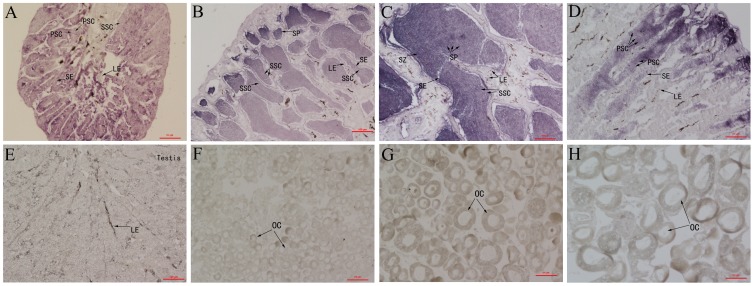
*In situ* localization of *tesk1* mRNA in gonads of *C. semilaevis*. Gonads *in situ* hybridization using antisense RNA probe of *tesk1* performed in tongue sole. (A): testis of diploid male at8 months, (B): testis of diploid male at 1 year, (C): testis of diploid male at 2 years, (D): testis of diploidpseudo-male at 2 years, (E): testis of triploid male at 2 years, (F): ovary of diploid female at 8 months, (G): ovary of diploid female at 1 year, (H): ovary of diploid female at 2years. Abbreviation of the cells indicated by arrows. PSC: primary spermatocyte, SSC: secondary spermatocyte, SP: spermatid, SZ: spermatozoon, SE: Sertoli cells, LE: Leydig cells, OC: oocyte. Scale bars, 10 µm.

*In situ* hybridization was used to determine which cells expressed *tesk1* mRNA in the testis. Intense hybridization signals were detected in both spermatids and spermatozoa (Fig. 4), while weak signals were detected in the secondary spermatocytes, and no signal was found in somatic cells. Paraffin sections of ovaries were also subject to *in situ* hybridization, but there was no evidence of a positive signal. Sense probes were used as a negative control (Fig. S2).

### Chromosomal localization of tongue sole *tesk1*

**Figure 5:**
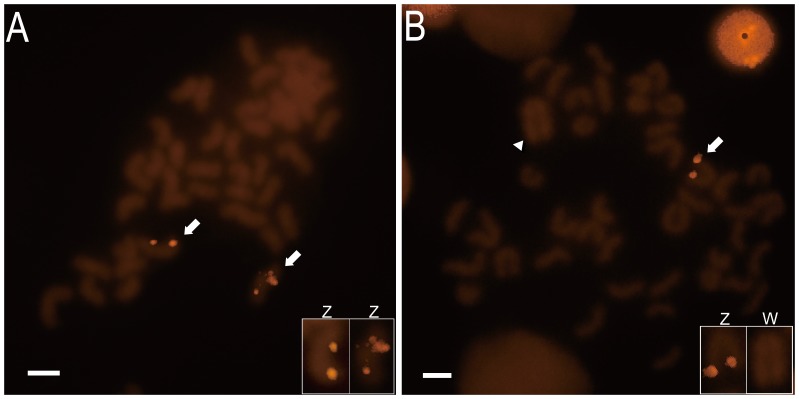
Chromosomal localization of the *C. semilaevis tesk1*. *Tesk1*-BAC FISH analysis of tongue sole chromosomes showing four signals in ZZ male (A) and two signals in ZW female (B). BAC clone Hind017D15, which contains the full length *tesk1* gene was labeled and used to probe male (ZZ) and female (ZW) chromosome spreads. Arrows indicate the fluorescent signals. Arrowhead indicates the W chromosome which is identified by its largest size of all chromosomes. Scale bars, 5 µm.

Four hybridization signals were observed on the metaphase chromosomes of ZZ males and two hybridization signals in ZW females (Fig. 5). Male individuals have two Z chromosomes whereas females have only one Z chromosome. Thus, our results suggest that the tongue sole *tesk1* gene is located on the Z chromosome.

## Discussion

We isolated and identified full length cDNA encoding the tongue sole Tesk1, a non-receptor type protein kinase. We also obtained the entire genomic sequence of *tesk1*. Tesk1 has significant homology with LIMK1 and LIMK2, two related members of a LIM motif-containing protein kinase subfamily [21], [22], [23], in the protein kinase domain. Phylogenetic analysis indicated that the Tesk1 of tongue sole has a very high similarity with that of other fishes such as *P. nyererei*, *M. zebra*, *O. niloticus*, and *S. salar*, as well as a high similarity with some species of reptiles and birds. Taken together, these observations suggest that *tesk1* has been highly conserved during evolution from fishes to birds. Interestingly, tongue sole Tesk1 shares a low level of similarity with mouse and human Tesk1.

Of the tongue sole tissues examined by qPCR, *tesk1* mRNA was detected mainly in the testis (Fig. 2A), indicative of an important role for Tesk1 in this tissue. This is consistent with previous observations in rats and mice [5]. A low level of *tesk1* mRNA expression was detected in the brain and other tissues, including the liver and kidney. Although we have no evidence to support the functional role of *tesk1* mRNA in the brain, we speculate that it might be involved with the hypothalamic-pituitary-gonadal axis. However, additional experiments are needed to confirm this.

We also evaluated the level of *tesk1* mRNA expression in the testes of individuals with different genotypes using real-time qPCR (Fig. 2B). *tesk1* mRNA expression was detected in the testes of male and pseudo-male individuals, but not in triploid males. Both male and pseudo-male tongue sole can produce normal sperm, whereas the development of the triploid male testis is inhibited such that it is unable to produce sperm [12]. Our findings therefore indicate that Tesk1 plays an important role in tongue sole spermatogenesis.

Spermatogenesis is a highly complex process consisting of three successively occurring events: spermatogonial mitotic renewal and differentiation into spermatocytes, the meiotic division of spermatocytes into spermatids, and spermiogenesis, a morphogenetic change of spermatids into highly differentiated spermatozoa [24], [25], [26], [27]. These events are thought to be regulated by numerous gene products at each stage of germ cell differentiation, and are synchronized in the same seminiferous tubule [28]. Gene knock-out mice have shown that a range of gene products expressed in testicular germ cells or somatic cells, including DNA repair enzymes, secretary factors, and transcription factors, are essential for male germ cell differentiation [29], [30], [31].

We detected *tesk1* mRNA expression in the tongue sole testis at different developmental stages. The level of expression between 16 and 116 days after hatching was very low, but this increased significantly in the testes of 5–8-month-old fry. At this point, the testis has completely differentiated following the completion of mitosis and the appearance of spermatocytes at 5 months, then the meiotic division of spermatocytes into spermatids at 8 months [10], [32]. *tesk1* mRNA expression level in the testes of 1-year old males had increased significantly. This coincides with a point in spermiogenesis during which spermatids change into spermatozoa via morphogenesis [10], [32]. The increase in *tesk1* transcription at these developmental stages suggests that it may have an important role during these processes, which is consistent with previous observations in rats [5].

*In situ* hybridization showed that the *tesk1* mRNA expressed in the testicular germ cells. This provides further support for our hypothesis that *tesk1* plays a role in spermatogenesis. We observed that *tesk1* mRNA is highly expressed at specific stages of spermatogenesis, particularly during the development of spermatids to spermatozoa. Based on this observation, we speculate that the primary function of Tesk1 in tongue sole is involved with spermatogenesis.

The *tesk1* gene was mapped to the tongue sole Z chromosome, which may explain the previous observations that W sperm was not produced [10]. Although the W chromosome is the largest one of all *C. semilaevis* chromosomes [9], it contains many redundant sequences so has fewer genes than the Z chromosome [10]. We speculate that the absence of *tesk1* in the W chromosome explains why W spermatozoa were not produced in pseudo-male tongue soles.

In summary, this is the first study of the *tesk1*gene in teleosts, and it reports the cloning of the full-length cDNA and genomic sequence of *tesk1* in *C. semilaevis*. *tesk1* mRNA was predominantly expressed in the testicular germ cells suggesting that Tesk1 plays an important role in spermatogenesis. However, the mechanism by which Tesk1 exerts its effects remains unclear and further study is warranted.

## Supporting Information

Figure S1 **Genomic sequence and decuced amino acid sequence of tongue sole** ***tesk1*** **gene.** Exons are in uppercase and introns are in lowercase. The stop codon is indicated by an asterisk. The terminal signals (AATAAA) in the 3′-untranslated region (UTR) are marked by grey box. Activiate sites of N-terminal kinase domain are marked by box, C-terminal proline-rich domain are underlined. A microsatellite site of (CT)_27_ located on the fourth intron is underlined by ∼∼.
(TIF)

Figure S2 ***In situ*** **hybridization of** ***tesk1*** **mRNA in gonads of** ***C.semilaevis*** **using sense probes.** (A): testis of diploid male at 8 months, (B): testis of diploid male at 1 year, (C): testis of diploid male at 2 years, (D): testis of diploid pseudo-male at 2 years, (E): testis of triploid male at 2 years, (F): ovary of diploid female at 8 months, (G): ovary of diploid female at 1 year, (H): ovary of diploid female at 2years. No hybridization signal was detected in all sections. Scale bars, 10 µm.
(TIF)

Table S1 **Primers and their sequences in this study.**
(DOCX)
