# Supplementary figures and images for: Cloning and Characterization of tesk1, a Novel Spermatogenesis-Related Gene, in the Tongue Sole (Cynoglossus semilaevis)
Source: PLoS One. 2014 Oct 1;9(10):e107922. doi: 10.1371/journal.pone.0107922 (PMC4182740; doi:10.1371/journal.pone.0107922)

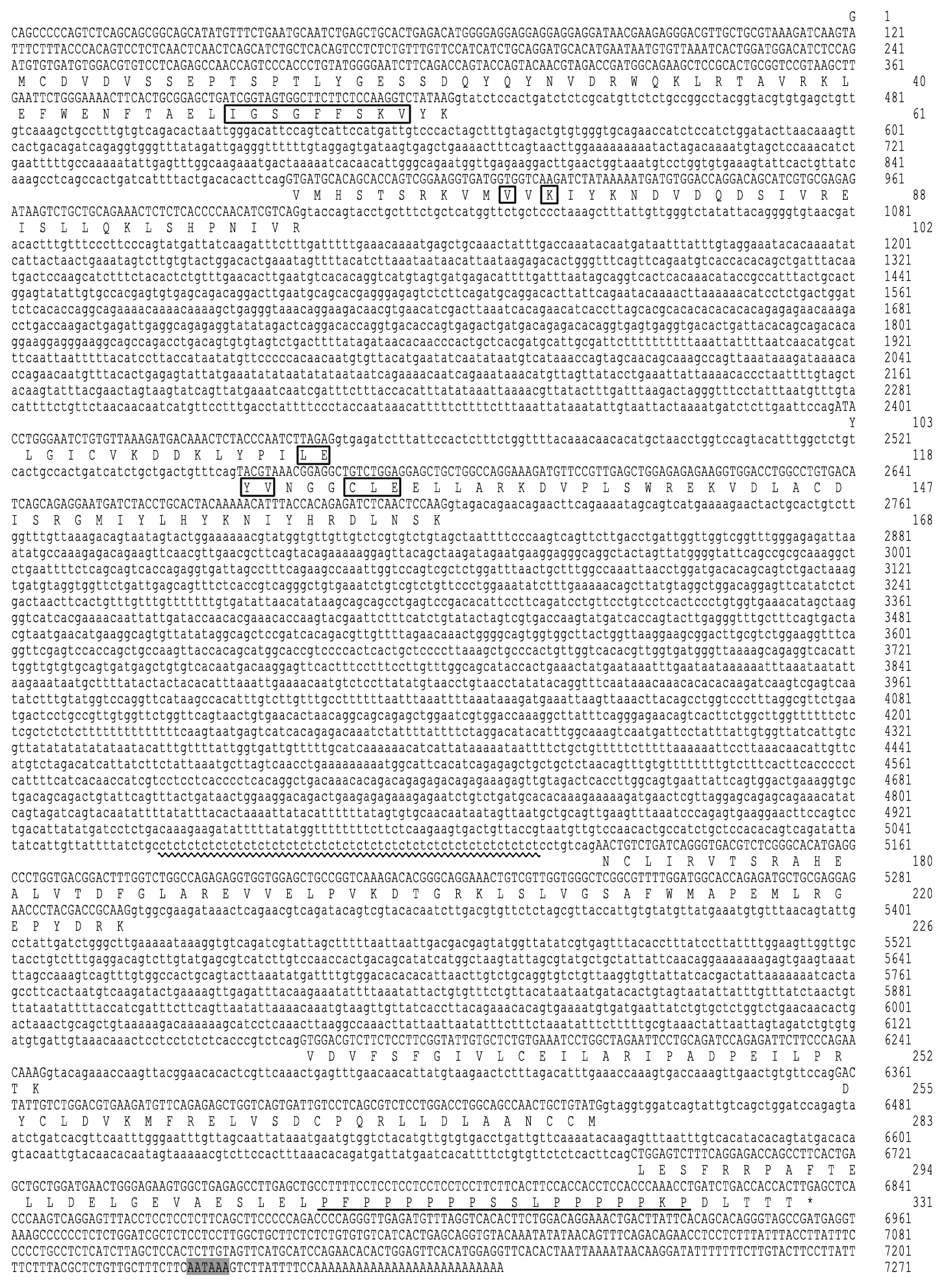

Supplement: Figure S1 — Genomic sequence and decuced amino acid sequence of tongue sole tesk1 gene. Exons are in uppercase and introns are in lowercase. The stop codon is indicated by an asterisk. The terminal signals (AATAAA) in the 3′-untranslated region (UTR) are marked by grey box. Activiate sites of N-terminal kinase domain are marked by box, C-terminal proline-rich domain are underlined. A microsatellite site of (CT)27 located on the fourth intron is underlined by ∼∼. (TIF) [file pone.0107922.s001.tif]

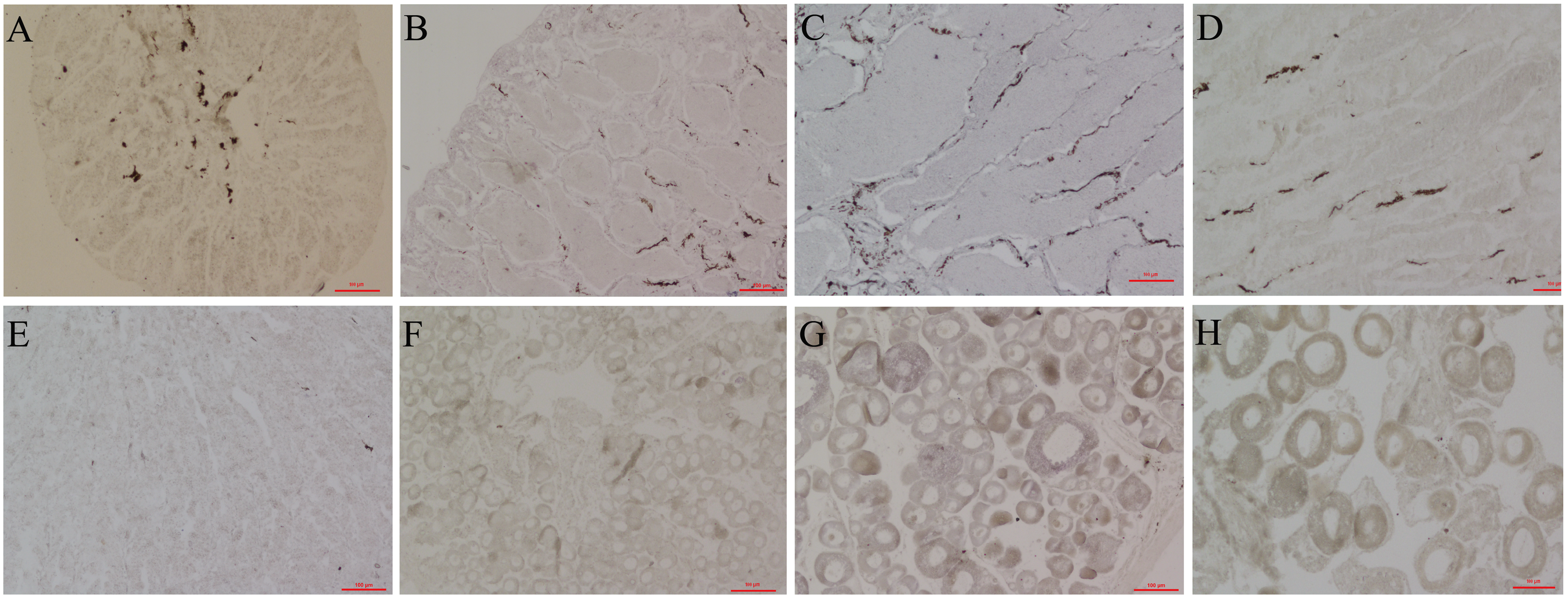

Supplement: Figure S2 — In situ hybridization of tesk1 mRNA in gonads of C.semilaevis using sense probes. (A): testis of diploid male at 8 months, (B): testis of diploid male at 1 year, (C): testis of diploid male at 2 years, (D): testis of diploid pseudo-male at 2 years, (E): testis of triploid male at 2 years, (F): ovary of diploid female at 8 months, (G): ovary of diploid female at 1 year, (H): ovary of diploid female at 2years. No hybridization signal was detected in all sections. Scale bars, 10 µm. (TIF) [file pone.0107922.s002.tif]
