# Supplementary material for: Cloning and Characterization of tesk1, a Novel Spermatogenesis-Related Gene, in the Tongue Sole (Cynoglossus semilaevis)
Source: PLoS One. 2014 Oct 1;9(10):e107922. doi: 10.1371/journal.pone.0107922 (PMC4182740; doi:10.1371/journal.pone.0107922)
Supplement: Table S1 — Primers and their sequences in this study. (DOCX) [file pone.0107922.s003.docx]

**Table S1**

Primers and their sequences in this study.

| Primer | Primer sequences (5’ to 3’) | Primer utilizations |
| --- | --- | --- |
| Cse-SSR1F | GAGGCCGACAGGATCGTAC | Genetic sex determination |
| Cse-SSR1R | TACGACGTACTCCGGTGGTTTT | Genetic sex determination |
| TESK3-1 | GGAAACTGTCGTTGGTGGGCTCG | 3’ RACE |
| TESK3-2 | GATGAACTGGGAGAAGTGGCTGAGA | 3’ RACE |
| TESK5-1 | GCCAGACCAAAGTCCGTCACCAGGG | 5’ RACE |
| TESK5-2 | GCTGTCCTGGTCCACATCATTTTTA | 5’ RACE |
| UPM long | CTAATACGACTCACTATAGGGCAAGCAGTGGTATCAACGCAGAGT | RACE |
| UMP short | CTAATACGACTCACTATAGGGC | RACE |
| NUP | AAGCAGTGGTATCAACGCAGAGT | RACE |
| TEG1S | GAGGATAACGAAGAGGGACG | genomic sequence amplification |
| TEG1A | CGATGTTGGGGTGAGAGAGT | genomic sequence amplification |
| TEG2S | GAAACTCTCTCACCCCAACA | genomic sequence amplification |
| TEG2A | CTCTCAGCCACTTCTCCCA | genomic sequence amplification |
| TEG3S | GTGGGCTCGGCGTTTTG | genomic sequence amplification |
| TEG3A | GATGAGAGGCAGGGGGAAAT | genomic sequence amplification |
| TE-RQ-S | GCAGAAACTCTCTCACCCCAACA | real-time quantitive RT-PCR |
| TE-RQ-A | CCAGACCAAAGTCCGTCACCA | real-time quantitive RT-PCR |
| Actin-S | GCTGTGCTGCTGTCCCTGTA | internal control |
| Actin-A | GAGTAGCCACGCTCTGTC | internal control |
| TESK-ISH-F | CGCGTCGACACAAAAACATTTACCACA | *in situ* hybridization |
| TESK-ISH-R | CGCGATATCACAGAGCACAATACCG | *in situ* hybridization |
